# What matters to you? Engaging with children in the James Lind Alliance Children’s Cancer Priority Setting Partnership

**DOI:** 10.1186/s40900-023-00518-2

**Published:** 2023-11-30

**Authors:** Susie Aldiss, Penelope Hart-Spencer, Loveday Langton, Sonia Malik, Keeley McEvoy, Jessica E. Morgan, Rosa Reed-Berendt, Rachel Hollis, Bob Phillips, Faith Gibson, the James Lind Alliance Children’s Cancer Priority Setting Partnership Steering Group

**Affiliations:** 1 School of Health Sciences, University of Surrey, Kate Granger Building, 30 Priestley Road, Surrey Research Park, Guildford, GU2 7YH UK; 2 The Christie NHS Foundation Trust, Manchester, UK; 3 Children’s Cancer Priority Setting Partnership Steering Group, London, UK; 4 Young Lives vs Cancer, London, UK; 5 Medical Needs Teaching Service, Leeds Children’s Hospital, Leeds, UK; 6 Centre for Reviews and Dissemination, University of York, York, UK; 7 Psychological and Mental Health Services, Great Ormond Street Hospital for Children, NHS Foundation Trust, London, UK; 8 Present Address: Leeds Children’s Hospital, Leeds, UK; 9 Hull-York Medical School, York, UK; 10 School of Health Sciences, University of Surrey, Guildford, UK; 11 Centre for Outcomes and Experience Research in Child Health, Illness and Disability (ORCHID), Great Ormond Street Hospital for Children NHS Foundation Trust, London, UK

**Keywords:** Children, Cancer, James Lind Alliance, Research priority setting, Patient and public involvement, Priority Setting Partnerships

## Abstract

**Background:**

Previous priority setting exercises have sought to involve children, but in the final reporting, it is evident that few children had been engaged through the process. A primary aim in the Children’s Cancer Priority Setting Partnership was to find out from children what they want research to focus on. We report on our experience to inform methods of engagement with children in future James Lind Alliance Priority Setting Partnerships and similar exercises.

**Methods:**

We followed the James Lind Alliance process, collecting and shortlisting questions via online surveys with adult survivors of childhood cancer, carers, and professionals, and holding a final workshop. Alongside this, a parallel process to collect and prioritise questions from children was undertaken. We created animations for parents/carers to explain the project and surveys to children, gathered questions via online surveys and held a workshop with children to identify their priorities.

**Results:**

Sixty-one children and young people with cancer and 10 siblings, aged 3–21 years, submitted 252 potential questions/topics via the surveys. Submissions were refined into 24 summary questions. These questions were discussed at a workshop with eight children; they also added more questions on topics of importance to them. Workshop participants prioritised the Top 5 questions; top priority was, ‘How can we make being in hospital a better experience for children and young people? (like having better food, internet, toys, and open visiting so other family members can be more involved in the child’s care)’. The Top 5 also included cancer prevention, treatments closer to home, early diagnosis, and emotional support. These questions were taken to the final workshop at which the Top 10 priorities were decided, all five children’s priorities were reflected in the final Top 10.

**Conclusions:**

We have demonstrated that it is possible to successfully involve children directly in setting priorities for future research. Future priority setting exercises on topics relevant to children, should seek to include their views. The Children’s Cancer Top 10 priorities reflect the voices of children and should inform the funding of future research.

**Supplementary Information:**

The online version contains supplementary material available at 10.1186/s40900-023-00518-2.

## Abstract (plain language summary)

Priority Setting Partnerships find out what areas of research are important to patients, families, and the professionals who care for them. Few Priority Setting Partnerships have involved children, so what matters to them may not have been well-represented. The Children’s Cancer Priority Setting Partnership aimed to find out directly from children what research we should do. We collected questions/topics for research from children using online surveys. We made animations to explain the project and surveys to children. Two-hundred and fifty-two questions were sent in by 61 children and young people with cancer and 10 siblings. We grouped similar questions together into 24 summary questions. Summary questions were discussed at a workshop with eight children. Workshop participants added more questions on topics that mattered to them, and decided their Top 5 questions. The top question was, ‘How can we make being in hospital a better experience for children and young people? (like having better food, internet, toys, and open visiting so other family members can be more involved in the child’s care)’. The Top 5 questions included: preventing cancer, having treatments nearer home, early diagnosis, and emotional support. These questions were taken to the final project workshop, this was with adults, including childhood cancer survivors, where the Top 10 priorities were decided. All five children’s priorities were included in the Top 10. We have shown it is possible to successfully involve children in setting research priorities. Future priority setting exercises on topics that affect children should actively seek and include their views.

**Supplementary Information:**

The online version contains supplementary material available at 10.1186/s40900-023-00518-2.

## Background

The James Lind Alliance (JLA) was established in the United Kingdom (UK) in 2004 to enable the end users of health research, patients, carers and professionals, to propose and agree on the most important topics in need of research [1]. Referred to as non-research stakeholders, this group has grown, with a review identifying 30 different stakeholder groups involved in priority setting projects [2]. It is consistently reported, however, that children, particularly younger children, are less represented in these exercises than other stakeholders [3, 4]. This is despite the fact that there is widespread support for children’s involvement in every phase of research [5]; beginning at the very first step of setting a research agenda. Engaging with stakeholders is well-recognised as challenging in terms of resources, capacity and feasibility [6]. Involving children is not straightforward [7]. We recognised the complexity, but at the outset of our research priority setting exercise we wanted to invest time, resources, and energy in anticipating and resolving any challenges that could impact on the participation of children, aged under 16 years, as non-research stakeholders.

Actively involving non-research stakeholders in setting a research agenda is enshrined in the processes and practices of the JLA methodology [1]. This change in direction, away from the traditional approach of researchers, research institutions or funding bodies deciding on the important questions to research, has been welcomed by some [8]. This is evidenced by the growing number of priority setting partnerships (PSPs) listed on the JLA website (https://www.jla.nihr.ac.uk/priority-setting-partnerships/) and by the creation of research funding calls directly answering questions from PSPs, such as from the National Institute for Health and Care Research (https://www.nihr.ac.uk/documents/nihr-james-lind-alliance-priority-setting-partnerships-rolling-call/28569). In these PSPs patients, carers, and healthcare professionals come together and, using a systematic approach, develop, and agree on shared priorities for research; the focus may be on questions relating to a particular health condition, a treatment uncertainty, or, in our case, a patient population [9]. We partnered with the JLA on the UK Children’s Cancer PSP. We wanted to find out from children, those under 16 years, what research should be done. We report here our approach to engaging with children in our PSP, and what they told us, making transparent how their voices were present in the outcome of this exercise, identifying the Children’s Cancer Top 10 priorities.

## Methods and results

**Fig. 1:**
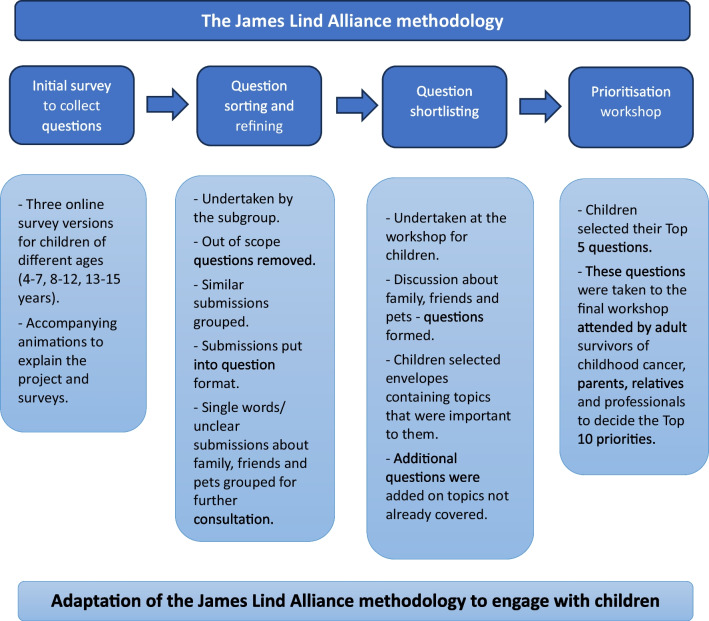
Overview of the James Lind Alliance methodology and how it was adapted to use with children

We aimed to conduct a UK-wide research prioritisation exercise for childhood cancer to inform decisions made by research funders and support the case for research in this underserved group; it is well described how little of the money raised is dedicated to children’s cancer [10]. Fundamental to this project, was our ambition to engage with children as key non-research stakeholders in the priority setting process. We followed the well-established JLA methodology, collecting and shortlisting questions via online surveys with adult survivors of childhood cancer, carers, and professionals, and holding a final workshop [1]. Alongside this, a parallel process to collect and prioritise questions from children was undertaken (Fig. 1). We report on this approach, our experiences of undertaking this work, and our findings, to inform future engagement with children in JLA PSPs and similar exercises. Our reporting was guided by the Guidance for Reporting Involvement of Patients and the Public (GRIPP2) short form [11], see Additional file 1.

### Project set up

#### Project management and steering group

There was a coordinating team of four researchers, nurses and clinicians. An expert steering group oversaw the whole project. The steering group included parents of a child with cancer (n = 5); an adult survivor of childhood cancer; a range of professionals reflecting the multidisciplinary nature of the care of children with cancer including: a teacher, General Practitioner, surgeon, pharmacist, dietitian, speech and language therapist, clinical psychologist, physiotherapist, nurses (n = 2), doctors (n = 6) and representatives from the third sector (n = 3), including the charities funding the project.

#### Scope

The project scope was agreed by the PSP steering group. Our focus was on cancer and cancer-like conditions in children aged 0 to 15 at initial diagnosis (up to their 16th birthday). This included survivors of childhood cancer who are now adults. The scope was kept intentionally broad and included questions on prevention, causes, diagnosis, treatment, care, follow-up, survivorship, relapse, and end-of-life care.

Our PSP aim was, *‘To identify gaps and unanswered questions in research about children’s cancer from patients, carers and professionals’ perspectives and then prioritise those that these groups agree are the most important for research to address’*.

In our previous Teenage and Young Adult Cancer PSP [12] few responses were received from those aged 13 to 15; therefore, the steering group decided to include this age group in the Children’s Cancer PSP. In the UK, children and young people’s cancer services serve patients from birth to 24 years. Children’s services are used by those between 0 and 16, and teenage and young adult services are used by those aged 16 up to their 25th birthday (there is some variation between particular centres).

#### Subgroup

A subgroup was established which included members of the coordinating team and project steering group who wanted to be involved in the PSP’s engagement with children. The subgroup consisted of two researchers, a teacher, medical doctor, health play specialist, parent, clinical psychologist, and charity representative.

### Gathering questions from children

At the start of our project, our plan was to run a series of face-to-face workshops with children to collect research questions and have each specialist children’s cancer treatment centre, known as Principal Treatment Centres in the UK (https://www.cclg.org.uk/In-hospital/Specialist-hospitals), involved in publicising the project to families within their care and helping to collect questions. These plans changed with the pandemic which meant that in-person group work was not possible until the final workshop in the PSP process. Drawing on experiences from other PSPs that had sought to involve children, face-to-face methods seemed to have been most successful [13, 14]. We learnt from the Learning Difficulties PSP [13], that some children can find it difficult to understand what is meant by research or how to phrase a question. Therefore, it was clear that we needed to help children to understand the process.

Following discussion with our subgroup, our next plan was for hospital school staff to work with children to complete the survey while they were in hospital or in the community. The teacher on our subgroup worked with other teachers to produce school lesson plans for children at different key stages in the national curriculum. The lesson content focused on explaining to children about research, engaging them in thinking about what matters to them and what questions about children’s cancer they would like to see answered by research, ending with completion of the survey. This approach was piloted in one cancer treatment centre, but it quickly became clear that this was not working. Although a few children did participate in the lessons and completed the survey, feedback from hospital school staff was that they were finding the lessons difficult to deliver, as there never seemed to be a good time. They felt that lessons in hospital were a time when children did not focus on their cancer and so asking them to think about their cancer experience and complete the survey did not feel appropriate. The subgroup decided that the best way to reach children would be through their parents/carers, with some additional support from professionals to promote the survey to families. We wanted to help parents to explain the project and survey to their child(ren). We thought that the use of age-appropriate animations would be a good way to do this.

We looked for an animator with previous experience of explaining research projects to children. One of our steering group members had worked previously with an animator from ScienceSplained (https://www.sciencesplained.com/). We decided to make two different animations, one for younger (https://www.youtube.com/watch?v=O492QZ1myko&t=72s) and one for older children (https://www.youtube.com/watch?v=pRaRuMr7ol0) that would allow children and families to self-select what looked most applicable to them. There was already an animation about the PSP process on the JLA website that was appropriate for teenagers (‘The PSP Process’ https://www.jla.nihr.ac.uk/about-the-james-lind-alliance/). The ideas for the animations were worked up by the subgroup along with the animator and the scripts were checked by children making sure that the ‘stories’ made sense to them. For further information about development of the animations see: https://www.jla.nihr.ac.uk/news/childrens-cancer-priority-setting-partnership-developing-animations-to-explain-the-jla-survey-to-children/28671.

Three different versions of the surveys were built using Qualtrics online software, aimed at children of different ages (4–7 years, 8–12 years and 13–15 years; available from: https://www.jla.nihr.ac.uk/priority-setting-partnerships/childrens-cancer/). Children were invited to complete whichever survey version they preferred. The surveys varied in the complexity of language used in the introduction section and questions, and the surveys for older children and teenagers contained more questions seeking demographic information. After discussion with parents on the PSP steering group, the word ‘cancer’ was not used in the survey or animation for younger children as they said that this would give flexibility for parents to use the words familiar to their own child when helping them to complete the survey. The surveys were piloted with children; no changes were suggested.

Surveys were launched on 6^th^ September 2021 and closed on 16^th^ November 2021 inviting children to participate who:

- were diagnosed with cancer before their 16th birthday;
- have a brother or sister with cancer now or who had cancer when they were younger (diagnosed before they were 16 years old);
- have a friend with cancer now or who had cancer when they were younger (diagnosed before they were 16 years old).

**Fig. 2:**
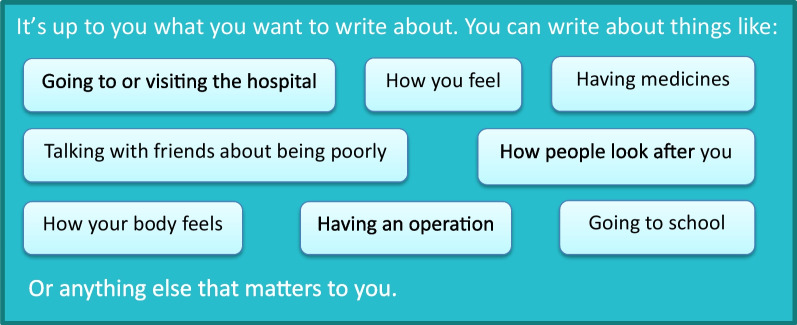
Topic suggestions from the survey (children aged 4–7)

Respondents were invited to submit up to eight questions/topics about any aspect of children’s cancer they considered important. Examples of topics that children may wish to consider were given on the question submission pages (Fig. 2). Surveys were promoted through the PSP’s Partner organisations (charities working with children with cancer and their families), social media and posters were sent to all Principal Treatment Centres.

### Children’s survey results

Seventy-four children and young people answered the surveys, three did not submit any questions and were removed from the analysis. A total of 71 respondents submitted 252 questions/topics. Sixty-one respondents were children and young people who had experienced cancer (aged 3–21) and ten were siblings (aged 4–19). Our surveys had been aimed at four to 15 year olds but as they were online, we could not restrict who completed them. We included responses from all participants in our analysis as our PSP scope encompassed survivors of childhood cancer who are now over 16 years, so these older respondents still fitted our inclusion criteria. No friends participated. See Table 1 for respondent demographics.

**Table 1:** Demographic details of children and young people who answered the survey

Response	Children and young people with cancer (n = 61)	Siblings (n = 10)
*Gender*		
Male	22 (36%)	5 (50%)
Female	38 (62%)	5 (50%)
Prefer not to answer	1 (2%)	0 (0%)
*Age*		
3–6 years	13 (21%)	1 (10%)
7–9 years	17 (28%)	2 (20%)
10–12 years	9 (15%)	2 (20%)
13–15 years	16 (26%)	3 (30%)
16–21 years	5 (8%)	1 (10%)
Prefer not to answer	1 (2%)	1 (10%)
*Country of residence*		
England	42 (69%)	6 (60%)
Scotland	9 (15%)	2 (20%)
Wales	6 (10%)	2 (20%)
Northern Ireland	1 (2%)	0 (0%)
Other	2 (3%)	0 (0%)
Prefer not to answer	1 (2%)	0 (0%)
*Diagnosis*		
Leukaemia	26 (43%)	3 (30%)
Kidney tumour	7 (11%)	0 (0%)
Lymphoma	7 (11%)	1 (10%)
Brain/spinal tumour	5 (8%)	2 (20%)
Soft tissue sarcoma	4 (7%)	0 (0%)
Neuroblastoma	3 (5%)	2 (2%)
Retinoblastoma	2 (3%)	0 (0%)
Bone tumour	1 (2%)	0 (0%)
More than one cancer diagnosis	1 (2%)	0 (0%)
Other	2 (3%)	1 (10%)
Prefer not to answer	2 (3%)	0 (0%)
Do not know	1 (2%)	1 (10%)
*Ethnic group*^*a*^* (children and young people with cancer n* = *36; siblings n* = *7)*		
White	31 (86%)	7 (100%)
Asian or Asian British	1 (3%)	0 (0%)
Black African, Black Caribbean or Black British	1 (3%)	0 (0%)
Mixed/multiple ethnic groups	1 (3%)	0 (0%)
Prefer not to answer	2 (6%)	0 (0%)
*Current situation*^*a*^* (children and young people with cancer n* = *36; siblings n* = *7)*		
On treatment	12 (33%)	3 (43%)
Finished treatment	23 (64%)	4 (57%)
Other	1 (3%)	0 (0%)

^a^Not asked in 4–7 year olds survey

### Analysis of questions from the children’s surveys

For brevity, we refer to submissions as ‘questions’; in reality, nearly all the submissions were not written as questions as respondents were invited to write what was important to them. All submitted questions were extracted from Qualtrics into an Excel spreadsheet. Multiple questions written in the same box were separated. In total, 252 questions were submitted. Thirty-four questions were from siblings.

An initial coding of the questions was carried out by coordinating team member SA, with support from FG. Questions were grouped into themes to make them easier to review and discuss. Themes were:

1. Impact on life
2. Treatment
3. Being poorly (unwell), side effects and long-term effects
4. Hospital experience
5. Emotional impact
6. Education
7. Family
8. Friends
9. Information and communication
10. Siblings

Some questions were coded in more than one category. Once all the questions had been coded, questions in the same category were grouped together and categories separated into different tabs within the Excel spreadsheet to assist with data management.

Thirteen questions were identified as out of scope and removed, as they were unrelated to cancer or were unclear, examples include, ‘cost to hospital’, ‘wildlife’ and ‘meeting new people’. These submissions were checked and exclusion agreed by subgroup members.

SA worked through the categories to further group similar questions together and form summary questions. The aim was to retain the sense of what the respondent meant, but in the form of a clear question. FG supported SA with this process.

The subgroup met online to review the summary questions with further checking undertaken via email until agreement was reached. This stage resulted in 24 summary questions. Many children responded that their family, friends, and pets were important to them, but it was unclear what it was about these aspects that were important (several responses were one or a few words, such as ‘family’, ‘mum and dad’, ‘seeing friends’). The subgroup decided that it would be wrong to guess or presume what children meant and further consultation with children was arranged. We planned to hold two online workshops for children to ask them about what was important to them about family, friends and pets but were unable to recruit enough participants to do this. Consequently, this discussion took place as part of the in-person workshop with children.

All survey submissions, with resulting summary questions are available from: https://www.jla.nihr.ac.uk/priority-setting-partnerships/childrens-cancer/

### Children’s workshop

The children’s workshop took place on Sunday 23rd October 2022 from 11 am to 3 pm. It was held in a community centre in central London and was facilitated by FG and SA. Recruitment to the workshop was via social media, through Principal Treatment Centres and emails to parents who had completed the PSP shortlisting survey and indicated they would like information on the children’s workshop. Eight children aged 8–16 attended; three were siblings. Their diagnoses included lymphoma and leukaemia; they were diagnosed between approximately one and 8 years ago. One participant was still on treatment. Parents were able to wait in the venue in a separate room if they wished, parents did not contribute to the discussion. Travel expenses were paid along with overnight accommodation for those who had far to travel. Each participant was given a £20 online voucher.

The workshop began with an ‘ice-breaker’ activity. We played ‘People Bingo’ to help everyone to get to know each other. Each participant (including the facilitators) was given a card with a 3 × 3 grid containing statements such as, ‘Someone who likes reading’, ‘Someone who has a pet’. The task was to go around the group and find someone who each statement applied to and write down their name against it.

**Fig. 3:**
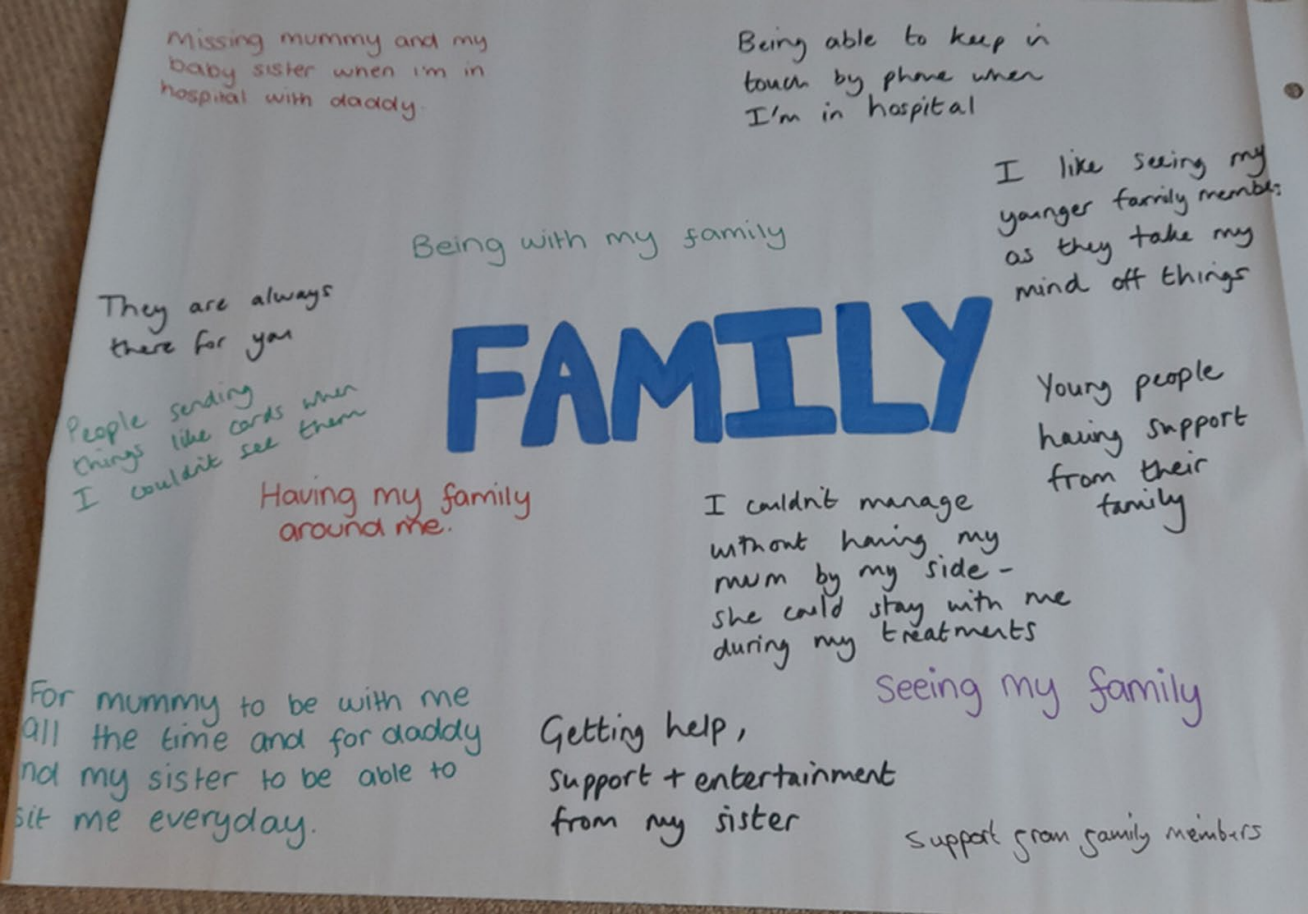
Photo from the discussion on what is important to children about ‘Family’

Once this activity was complete, the facilitators gave a brief introduction to the day, outlining the purpose of the workshop and what was going to happen. We then moved on to a discussion about ‘family, friends, and pets’, to make some summary questions on these topics as the meaning of the submissions to the survey about these aspects had been unclear. The words ‘family’, ‘friends’ and ‘pets’ were each written in the centre of a sheet of A1 size flip-chart paper. The submissions from the surveys about each topic were written around the word. Each topic was discussed in turn, this focused on what was important to the participants about family, friends, and pets—their responses were added to the paper by one of the facilitators (Fig. 3).

Each topic was then revisited; the facilitators gave a verbal summary of what was on each sheet and worked with the children to support them to make summary questions for each topic. Seven summary questions were created (see question numbers 3 to 9 in Table 2). Each question was written onto a card in preparation for the next discussion.

**Table 2:** Summary questions in the envelopes at the children and young people’s workshop

No	Topic/summary question
*Family, friends and pets*	
1	How can we make sure children and young people can still do the things they want to do with their friends and family (like playing sports and going on holiday)?
2	What can hospital staff do to make sure children and young people feel involved when their brother or sister is in hospital?
3	How can we make the most of open visiting so other family members can be more involved?^a^
4	How can we make sure all children and young people can see all family members when they are hospital?
5	What are the best ways to spread awareness to help friends and classmates understand the reality of cancer?
6	How can we help children and young people to stay connected with friends and keep their relationships strong during treatment and afterwards?
7	What are the best ways to help children and young people to keep in contact with family and friends when they are in hospital?
8	How can we make it so children can meet and interact with their pets when they are in hospital?
9	How can we help more children to see therapy animals when they are in hospital?
*Treatments and medicines*	
10	How can we make the experience of having injections (needles) better for children and young people?
11	How can we make the experience of taking medicines better for children and young people? (including having the choice of tablets or liquid medicine)
12	How can we help children and young people to have as much time at home as possible instead of being in hospital?
13	What treatments work the best for children and young people to make them better?
*Being poorly, side effects and long-term effects*	
14	How can we help children and young people when treatment changes the way they look? (like when they lose their hair)
15	What helps children and young people when they are feeling poorly?
16	Can we find ways to use treatments that make children and young people feel less poorly? (like feeling less sick, not feeling weak)
*Being in hospital*	
17	How can we make being in hospital a better experience for children and young people? (like having better food, internet, visitors, toys)
18	How can play and distraction help children and young people with procedures? (like having something else to think about or do when having scans and cannulas)
19	What can hospital staff do that helps children and young people to feel well looked after in hospital?
*Emotions, worries and getting help or support*	
20	What are the best ways to help children and young people with their worries and make them feel happier?
21	How can we help and support children and young people after treatment has finished?
22	What are the best ways to help families with their worries when a child or young person is poorly? (including brothers, sisters, parents and grandparents)
23	How can we help children to meet other children and young people who are poorly like them?
*School and education*	
24	How can we help children and young people to go to school or nursery during and after treatment?
25	How can we help children and young people to keep up with schoolwork when they are poorly or in hospital?
*Getting the information you need*	
26	How can we give children and young people the information they want about their illness and treatment in a way that they understand it?
27	What do children and young people need to know about who can help them and their family? (including how charities can help them)
28	How can hospital staff help children and young people to be involved in decisions about them in the way they want to be?
29	How can we help children and young people to talk with their friends and family about their illness?
30	How can we help people to understand about disabilities that you can’t see?
31	How can we give children and young people the information they want about their brother or sister’s illness and treatment in a way that they understand it?

^a^This question was later combined with, ‘How can we make being in hospital a better experience for children and young people? (like having better food, internet, visitors, toys)’ to make: ‘How can we make being in hospital a better experience for children and young people? (like having better food, internet, toys, and open visiting so other family members can be more involved in the child’s care)’

We then followed the methodology used by the Children’s Arthritis PSP in the Netherlands [15]. We had seven envelopes, each containing questions on a different topic. In total there were 31 questions—24 summary questions from the children’s surveys (Table 2), plus the seven new questions on family, friends, and pets. The topics were:

1. Family, friends, and pets
2. Treatments and medicines
3. Being poorly, side effects and long-term effects
4. Being in hospital
5. Emotions, worries and getting help or support
6. School and education
7. Getting the information you need.

**Fig. 4:**
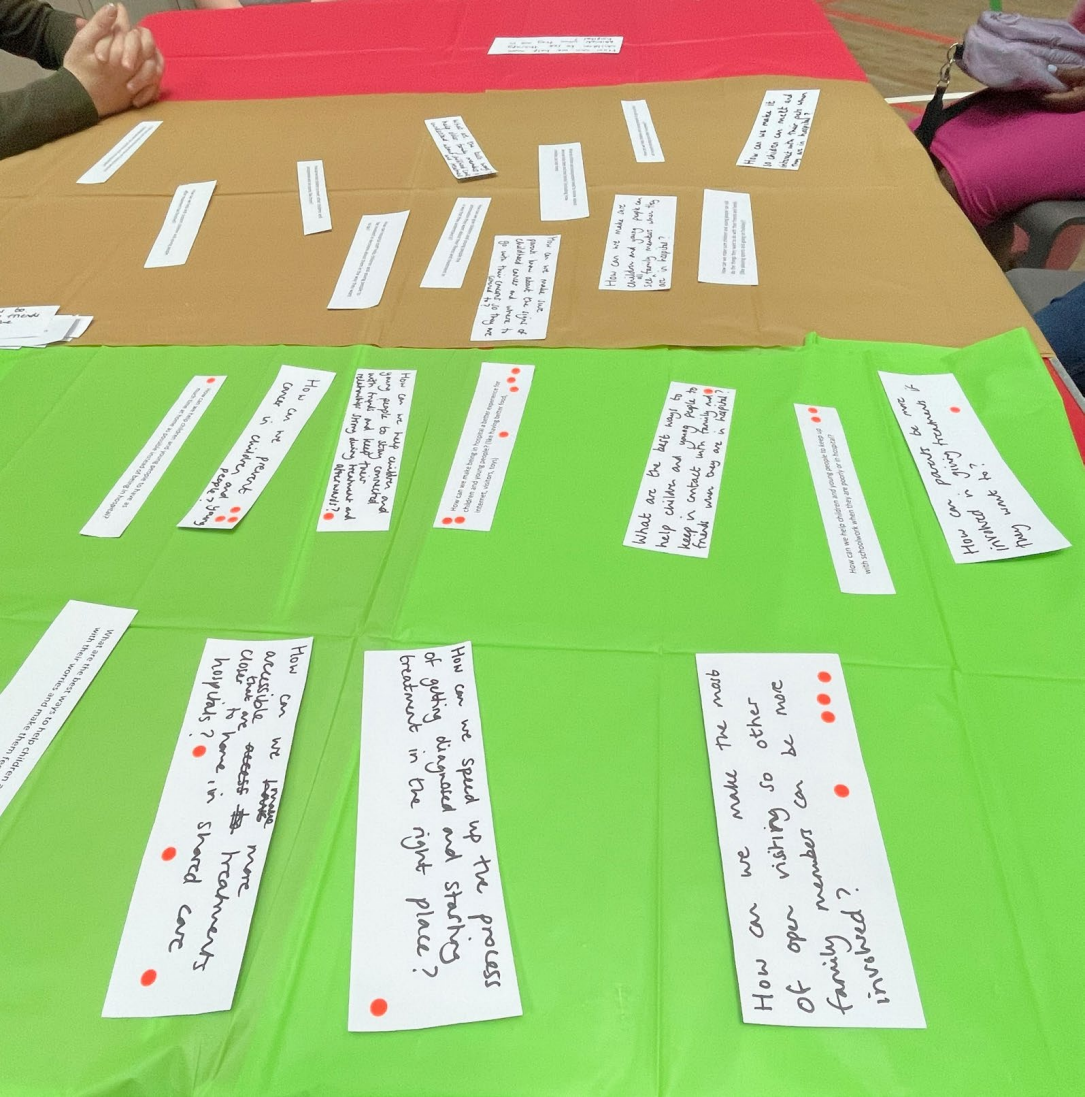
Photo from the children’s workshop showing the ranking system with coloured tablecloths

Each participant chose an envelope that corresponded to a topic that was important to them. They could share envelopes if they wished; six participants worked in pairs and two worked individually. Topics not picked were ‘Being poorly, side effects and long-term effects’ and ‘Being in hospital’. The table was covered in red, amber, and green tablecloths (Fig. 4). These tablecloths are often used in JLA workshops to help participants order the questions [1].

The colours represented the importance of the question; green was most important, red was least important, with amber being medium importance. The envelopes were opened, and the participants placed the questions on the table in the colour that reflected the importance of the question to them. Only one question was placed in the red area (How can we help more children to see therapy animals when they are in hospital?). Participants looked at the questions on the table and were invited to add more questions if there was anything missing that was important to them. Six questions were added (see Table 3) and were written onto cards. The participants decided together in which section each of these new questions should be placed.

**Table 3:** Additional questions added by children at the workshop

1	How can we prevent cancer in children and young people?
2	How can we make more accessible treatments that are closer to home, in shared care hospitals?
3	How can we speed up the process of getting diagnosed and starting treatment in the right place?
4	How can we make sure parents know about the signs of childhood cancer and where to go with their concerns, so they are listened to?
5	What are the best ways to help older family members to understand about childhood cancer and treatments?
6	How can parents be more involved in giving treatments if they want to?

Improving the experience of being in hospital was also discussed, and they wanted to create a question about this. There was already a question on this topic in the envelope on ‘Being in hospital’ which had not been opened. The participants were shown the question, ‘How can we make being in hospital a better experience for children and young people? (like having better food, internet, visitors, toys)’, they decided that it reflected what they wanted to say and placed it in the green area.

Each participant was given three stickers which they could use to vote for their Top 3 questions in the green area. Before the voting took place, they could move any questions from the amber/red areas up if they wished, so that they could be included in the vote. The questions were then placed in order of most to least votes and a discussion followed to agree the ‘Top 5’. We were again guided by the Children’s Arthritis PSP in the Netherlands, who had recommended that a Top 5, rather than a Top 10 works better with children as it shortens the discussion process [15]. The question, ‘How can we make being in hospital a better experience for children and young people? (like having better food, internet, visitors, toys)’ had received six votes and was placed as top priority. Underneath this was, ‘How can we make the most of open visiting so other family members can be more involved?’, which had four votes. Many of the participants at the workshop had experienced restricted visiting during the pandemic; they spoke about only being able to have one parent with them in hospital, and not being able to have siblings, extended family, and friends to visit which had been difficult for them. The children discussed how important it was for family members to be able to visit them in hospital as this helped them to understand more about the treatment. These relatives could also offer support to the child and their parents/family and be involved in the child’s care. As the question about improving hospital experience already mentioned ‘visitors’, the participants asked if they could expand on this to combine it with the question about open visiting which would then leave room for another question on a different topic in the Top 5. The question was changed to: ‘How can we make being in hospital a better experience for children and young people? (like having better food, internet, toys, and *open visiting so other family members can be more involved in the child’s care*)’.

The question, ‘How can we prevent cancer in children and young people?’ was placed at number 2 as the participants said that if cancer could be prevented, then answering the other questions would not be necessary. ‘How can we make more accessible treatments that are closer to home, in shared care hospitals?’ was placed at number 3 as some participants had to travel a long way for treatment and they wanted treatments to be available closer to home. This question, ‘How can we help children and young people to keep up with schoolwork when they are poorly or in hospital?’ was initially in the Top 5 but was then moved out as the participants discussed that it was possible to catch up with schoolwork later and they did not always feel like doing schoolwork when they were very unwell; the questions about getting a timely diagnosis and being supported emotionally were considered more important.

The Top 5 are shown in Table 4. Three of the questions were closely aligned to the questions already being taken to the final workshop from the shortlisting survey (priorities 2, 4 and 5). For priority 4, the children’s version of the question had an extra part on the end about starting treatment in the right place, therefore their version of the question was taken to the final workshop rather than the version from the shortlisting survey. Priorities 1 and 3 from children were new and were added into the list of questions for the workshop, making 23 questions for the final workshop in total.

**Table 4:** Children’s Top 5 and questions for the final workshop

Rank	Top 5 questions from the children’s workshop	Question for the final workshop from the shortlisting survey
1	How can we make being in hospital a better experience for children and young people? (like having better food, internet, toys, and open visiting so other family members can be more involved in the child’s care)	
2	How can we prevent cancer in children and young people?	Why do children develop cancer (including the role that genetics plays) and could it be prevented?
3	How can we make more accessible treatments that are closer to home, in shared care hospitals?	
4	How can we speed up the process of getting diagnosed and starting treatment in the right place?	How can time to diagnosis be improved for children with suspected cancer?
5	What are the best ways to help children and young people with their worries and make them feel happier?	What are the best ways to provide emotional support for children and their families (1) around the time of diagnosis, (2) during treatment and (3) after treatment (including survivors who are now adults)?

### Top 10 priorities for children’s cancer

Following the JLA process, the last stage, was a final prioritisation workshop. This took place in central London on 1st November 2022 to identify the Top 10 unanswered questions for future research on children’s cancer. The workshop was attended by 25 participants: four young adults who had experienced childhood cancer, five parents and one grandparent of a child who had experienced cancer, and 15 professionals who work with children who have cancer and their families. Discussions to decide the Top 10 took place in three groups over the course of the workshop, with the group coming together at the end to agree the final order. The participants were clear that they wanted to ensure the Top 10 questions included most, if not all, of the questions that were in the children’s Top 5. Following an initial discussion and ordering of questions, the discussion groups were told which questions were important to children (this was indicated on the back of the question cards). Those question cards were picked out and moved high up the ranking and their placement discussed. Most of these questions remained ranked in the Top 10, or just outside, for the duration of the discussions. There was a lot of discussion about whether the question, ‘What are the best ways to provide emotional support for children and their families: (1) around the time of diagnosis, (2) during treatment and (3) after treatment (including survivors who are now adults)?’ should be in the Top 10, as the question about emotional support from children was originally mapped onto this question. The question ended up at number 11 as the workshop participants decided that priority 3, ‘Are the psychological, practical, and financial support needs of children with cancer, survivors, and their families being met during treatment and beyond? How can access to this support be improved and what further support would they like?’ was related to the children’s original question as it includes emotional support as well as other types of support. All five of the top priorities identified by children are reflected in the final Top 10 (Table 5).

**Table 5:** Top 10 research priorities for Children’s Cancer

1	Can we find effective and kinder (less burdensome, more tolerable, with fewer short and long-term effects) treatments for children with cancer, including relapsed cancer?
2	Why do children develop cancer (including the role that genetics plays) and could it be prevented?^a^
3	Are the psychological, practical, and financial support needs of children with cancer, survivors, and their families being met during treatment and beyond? How can access to this support be improved and what further support would they like?^b^
4	How can we speed up the process of getting diagnosed and starting treatment in the right place?^a^
5	Why do children relapse, how can it be prevented, and what are the best ways to identify relapse earlier?
6	How can we make being in hospital a better experience for children and young people? (like having better food, internet, toys, and open visiting so other family members can be more involved in the child’s care)^a^
7	What are the best ways to ensure children and families get and understand the information they need, in order to make informed decisions, around the time of diagnosis, during treatment, at the end of treatment and after treatment has finished?
8	What impact does cancer and treatment have on the lives of children and families after treatment, and in the long-term; what are the best ways to help them to overcome these impacts to thrive and not just survive?
9	How can we make more accessible treatments that are closer to home, in shared care hospitals?^a^
10	What is the relationship between chronic fatigue syndrome, fibromyalgia, chronic pain and treatment for childhood cancer? (Fibromyalgia is a long-term condition that causes pain all over the body)

^a^These questions were in the Top 5 research priorities identified by children

^b^This question was originally not mapped onto the question about emotional support from children, but the workshop participants decided that this question was related as it includes emotional support as well as other types of support

## Discussion

From the outset of the Children’s Cancer PSP, the steering group were determined that the voices of children would be heard; that they might set research agendas and participate in more equal ways [16]. Previous PSPs have reflected that they were unable to engage with children as they wished, due to lack of time and resources [17]. A review focusing on research priority setting in paediatric long-term conditions published in 2018 reported that, in the 83 studies included, about a quarter reported parental/caregiver involvement, and only four involved children directly [18]. This is gradually changing, with a more recent review by Postma et al. identifying a trend in growth in the involvement of children and young people in setting research agendas [4]. Postma et al.’s review included 22 projects of priority setting exercises with children and young people aged 6–25 years, with 16 exercises using the JLA approach. The number of children and young people involved in the included projects ranged from 1 to 108, with a tendency for very few children, particularly younger children, to be involved. In the reporting of these priority setting exercises, it was unclear what is important to child versus adult participants and the impact of involving children was not discussed [4].

At the outset of developing our Children’s Cancer PSP we invested time and resources to engage with children and, on reflection, we would consider this aspect of our PSP a success, we heard from a larger group of children than many other PSPs with 71 children and young people sending in 252 questions/topics for research via our surveys. We also heard from some very young children, the youngest child to complete the survey was 3 years of age and 14 children who took part were aged 3–6 years. Our approach in undertaking a separate process with children has enabled us to hear what is important to them and how this compares to the ‘adults’ involved in the PSP. Overall, questions from children reflected similar themes to those from adult participants, such as support during and after treatment, improving experiences of treatment, reducing side effects and provision of information about cancer and treatment. There were some additional elements that featured as higher priority for children, such as having treatments closer to home and improving the hospital experience. Being able to have family and friends visit them in hospital was high priority for these children, reflecting their experiences during the pandemic when visiting was restricted.

At the final workshop, all participants paid attention to the perspectives of children and were in agreement that their voices should be heard as important stakeholders in this priority setting agenda. This resulted in all five of the top priorities identified by children being reflected in the final Top 10. Our approach of establishing the children’s top priorities first allowed the voices of children to influence the decision making of the adults at the final workshop. Other approaches, such as giving the children the priorities that adults had decided on to discuss or trying to integrate two separate lists of children’s and adults’ priorities, may have led to different outcomes where the voices of children were not as prominent. We hope that being able to see the priorities were also important to children with cancer will add extra ‘weight’ when future funding decisions are being made and research projects are being developed.

Engaging with children took both flexibility, in adapting our approach, and careful planning. We had to ensure our methods were accessible and appropriate. Much is written about how to work with children, in the context of research, and being creative in constructing knowledge with them [19], we drew heavily on work such as this. Early on in our PSP, the steering group recognised that engaging with children required dedicated focus and the subgroup was formed to oversee this aspect of the PSP; this was our first important step, the subgroup’s role was to ensure we came up with strategies to maximise children’s competencies and strengths to participate. We had included additional funds to engage with children in our original project planning—this had been for researchers to undertake face-to-face group work to collect research questions, similar to how other priority setting exercises have worked to involve children [15, 20]. This face-to-face work was not possible due to pandemic restrictions. We then tried a one-to-one in-person method with children, working with hospital school staff who were still seeing children face-to-face. We have reported here that this did not work, we listened to the feedback from school staff trying to deliver the sessions with children that it was not appropriate to engage with children during a time in hospital that allowed some ‘normality’ for them and was not focused on their cancer and treatment; this feedback was so important in guiding our approach.

Instead, we used some of the project funds to produce animations. We had learned from the Learning Difficulties PSP [13] that it is not always easy for children to understand what is meant by research or how to phrase a question. In the Learning Difficulties PSP, they showed as examples a list of existing questions that had been submitted by parents/carers and professionals. The language of these questions was adapted to be child-friendly. These accommodations helped children to complete the questionnaire with the help of the Occupational Therapists and Speech and Language Therapists. This reinforced to us the need to find ways to help children understand what the process is, what is meant by research, and research questions, and that children might need some help to complete the surveys. As we were not able to work with children face-to-face, we recognised that parents/carers could assist children in the process. The animations were designed for parents/carers and children to watch together and to help parents/carers to support their child to complete the survey. We do not know how successful these animations were in helping parents/carers and children when completing the survey as we do not have feedback on this. In their review, Odgers et al. reported that even though studies seeking children and young people’s perspectives used surveys written in plain, non-technical language, a large number of out-of-scope responses were received [16]. In our PSP, the high number of responses from children compared to similar PSP surveys, and the few ‘out-of-scope’ submissions from children might suggest that the animations were helpful. We did still receive some single/few word responses but with further consultation at the workshop, we were able to develop these into questions. These short, ambiguous responses are not limited to undertaking surveys with children, such responses are also often submitted by adults to PSP surveys [21].

### Strengths and limitations

One of the strengths of our project was the subgroup which brought together people with skills and experience in methods of engaging and communicating with children, either in research or other roles (e.g. teacher, health play specialist, parent). Inclusion of experts from beyond the field of healthcare enabled us to consider wider routes to support children to give their views (such as the school setting). We were able to take the well-established JLA process, involving the use of surveys and a workshop, and adapt it to work with children. We learnt from the successful approach of the Children’s Arthritis PSP and made adaptions where they had reported challenges. For example, in their priority setting workshop with children, it was reported that the participants found it hard to ‘let go’ of their personal priorities to come to a consensus [15]. This was not an issue in our workshop, the participants were quickly able to reach an agreement on the final Top 5 priorities. The additional step we added of each participant voting for their top three questions may have helped with ordering the priorities into a Top 5 and reaching consensus.

Absent voices must be considered as a limitation of this PSP. Of note, the majority of participants described themselves as White. The priorities therefore represent the views of the majority, White population, which has been observed in other PSPs [12, 22]. Although siblings were included in the workshop, we had a low number of responses from them in the survey. We also did not have any friends of children with cancer participate in the survey, which was a group we had sought to include.

### Recommendations for future priority setting exercises involving children

From our experience of undertaking a priority setting exercise with children, there are some recommendations we would like to share.

Surveys:

- Develop a subgroup focused on gathering data from children—this was important for us to prioritise children’s involvement in the best ways possible.
- Involve teachers, play specialists, and others who work closely with children in a learning capacity in the subgroup—this was essential for us to draw on the skills of a range of people who worked with children with cancer in different roles.
- Involve parents in the subgroup—their knowledge of their child with cancer helped us to keep a focus on the practical elements and how the survey would work.
- Have different versions of surveys for different ages—it was important we could offer choice, letting children decide which survey they completed.
- Ensure surveys are reviewed by children who are similar to the children who might complete the surveys—we were lucky to be able to gain feedback from children through a hospital school as we had involved a teacher in our PSP.
- Use animations in the process—we used animations to explain what the survey was asking children to do; animations can be an engaging way to give children information.
- Keep surveys simple and short—to encourage children to participate, ensuring the surveys are not a burden to complete.
- Use an online survey—this worked well and meant easier recruitment and reach than more traditional approaches.

Workshop:

- Allow some time at the start of the workshop for participants to get to know each other—we used an ‘ice-breaker’ task which also involved moving around the room so that participants were not sitting for too long and this worked well.
- Create an informal atmosphere where participants can openly share their thoughts—this is always important, but this was easier for some participants than others, with some children needing extra support to join the group conversation.
- Have three facilitators for the workshop—we had aimed to have a third facilitator, a teacher, who was unfortunately unable to attend on the day. This additional person would have been able to give more to support to participants who did not find it so easy to speak in a group situation. Having a third facilitator present would have given more flexibility in how we involved the participants, for example a few may have preferred to do some one-to-one work/smaller group work and then feed their ideas back to the whole group.
- Ask parents beforehand about whether their child has any additional needs—this enabled us to meet needs to support participation, such as help with reading or writing. We were aware of anyone needing this support in advance and were able to sensitively offer help with tasks such as reading through the questions in the envelopes.
- Keep close to the JLA principles and use their techniques where possible—this allows us to build a method that can be used with children in other PSPs.

## Conclusions

The Children’s Cancer Top 10 priorities include the voices of children with cancer, their families, survivors of childhood cancer and professionals. The uncertainties identified are the outcome of a systematic and transparent process and provide funders with clear guidance on the highest priorities for future research, voted on by end-users of research. It is now critical that funders and researchers take note of these shared priorities to ensure future research focuses on what is important to these stakeholders [23]. We have demonstrated that, with both flexibility and careful planning, it is possible to successfully involve children in setting priorities for future research on health conditions that affect them. One of the seven principles of a child’s rights-based approach states: all children have the right to have a say in matters that affect them and to have their views taken seriously (https://www.unicef.org.uk/child-friendly-cities/crba/). We prioritised giving space to children to express their views, drawing on our shared knowledge and experience that values children’s agency [24]. Children have expressed their views, now funders and researchers must consider these views, and mirror the participants in our final workshop where much weight was given to their priorities.

Future priority setting agendas on topics relevant to children should seek to include their views, in all stages of the process, from identifying questions/topics of importance, through to prioritisation. Along with national and international colleagues who have engaged with children in similar processes, we are working with the JLA to co-produce guidance on involving children in PSPs. Our aim is to champion methodological approaches to the involvement of children in research priority setting exercises.

### Supplementary Information

**Additional file 1:** **GRIPP2 short form checklist.**

## Data Availability

The data from the surveys generated and analysed during the current study are available from: https://www.jla.nihr.ac.uk/priority-setting-partnerships/childrens-cancer/.

## Abbreviations

GRIPP: Guidance for Reporting Involvement of Patients and the Public
JLA: James Lind Alliance
PSP: Priority Setting Partnership
UK: United Kingdom
