# Supplementary material for: What matters to you? Engaging with children in the James Lind Alliance Children’s Cancer Priority Setting Partnership
Source: Res Involv Engagem. 2023 Nov 30;9:110. doi: 10.1186/s40900-023-00518-2 (PMC10688066; doi:10.1186/s40900-023-00518-2)
Supplement: Supplementary file 1 — Additional file 1: GRIPP2 short form checklist. [file 40900_2023_518_MOESM1_ESM.docx]

**Additional File 1**

**GRIPP2 Short Form Checklist**

From: Staniszewska, S., Brett, J., Simera, I. et al. GRIPP2 reporting checklists: tools to improve reporting of patient and public involvement in research. Res Involv Engagem 3, 13 (2017). <https://doi.org/10.1186/s40900-017-0062-2>

| **Section and topic** | **Item** | **Reported on page No** |
| --- | --- | --- |
| 1: Aim | Report the aim of PPI in the study | End of background section and start of methods and results section |
| 2: Methods | Provide a clear description of the methods used for PPI in the study | Gathering questions from children and young people section and Children and young people’s workshop section |
| 3: Study results | Outcomes—Report the results of PPI in the study, including both positive and negative outcomes | Children and young people’s survey results section and  Children and young people’s workshop section. |
| 4: Discussion and conclusions | Outcomes—Comment on the extent to which PPI influenced the study overall. Describe positive and negative effects | Top 10 Priorities for Children’s Cancer and Discussion section. |
| 5: Reflections/critical perspective | Comment critically on the study, reflecting on the things that went well and those that did not, so others can learn from this experience | Included in Methods and Results section and in the Discussion and Conclusion. |
